# How bumblebees coordinate path integration and body orientation at the start of their first learning flight

**DOI:** 10.1242/jeb.245271

**Published:** 2023-04-21

**Authors:** Thomas S. Collett, Theo Robert, Elisa Frasnelli, Andrew Philippides, Natalie Hempel de Ibarra

**Affiliations:** ^1^ School of Life Sciences, University of Sussex, Brighton, BN1 9QG, UK; ^2^ Centre for Research in Animal Behaviour, Psychology, University of Exeter, Exeter, EX4 4QG, UK; ^3^ School of Engineering and Informatics, University of Sussex, Brighton, BN1 9QJ, UK

**Keywords:** First fixation of nest, Preferred viewing direction, Translational scan

## Abstract

The start of a bumblebee's first learning flight from its nest provides an opportunity to examine the bee's learning behaviour during its initial view of the nest's unfamiliar surroundings. Like many other hymenopterans, bumblebees store views of their nest surroundings while facing their nest. We found that a bumblebee's first fixation of the nest is a coordinated manoeuvre in which the insect faces the nest with its body oriented towards a particular visual feature within its surroundings. This conjunction of nest fixation and body orientation is preceded and reached by means of a translational scan during which the bee flies perpendicularly to its preferred body orientation. The utility of the coordinated manoeuvre is apparent during the bees' first return flight after foraging. Bees then adopt a similar preferred body orientation when close to the nest. How does a bee, unacquainted with its surroundings, know when it is facing its nest? A likely answer is through path integration, which gives bees continuously updated information about the current direction of their nest. Path integration also gives bees the possibility to fixate the nest when their body points in a desired direction. The three components of this coordinated manoeuvre are discussed in relation to current understanding of the central complex in the insect brain, noting that nest fixation is egocentric, whereas the preferred body orientation and flight direction that the bee adopts within the visual surroundings of the nest are geocentric.

## Teaser

**Summary:** Bumblebees fixate the nest while viewing a preferred location in their visual surroundings. This conjunction is aided by a preceding translational scan.

## INTRODUCTION

When ants, bees or wasps of many species first leave their nest, they perform, in its immediate vicinity, what have come to be called ‘learning walks’ for ants and ‘learning flights’ for bees and wasps (for reviews, see Collett and Zeil, 2018; Zeil and Fleischmann, 2019; Zeil et al., 1996). The insects' learning behaviour consists of an intricate path during which an insect periodically turns back to face its nest. Nest facing is crucial as it is then that an insect most probably learns the visual surroundings of the nest (Bates, 1863; Lehrer, 1993). A view recorded when facing the nest enables a returning insect to use that memory to guide its return to the nest (Dewar et al., 2014; Hempel de Ibarra et al., 2009; Robert et al., 2018; Zeil, 1993b).

**Fig. 1.**
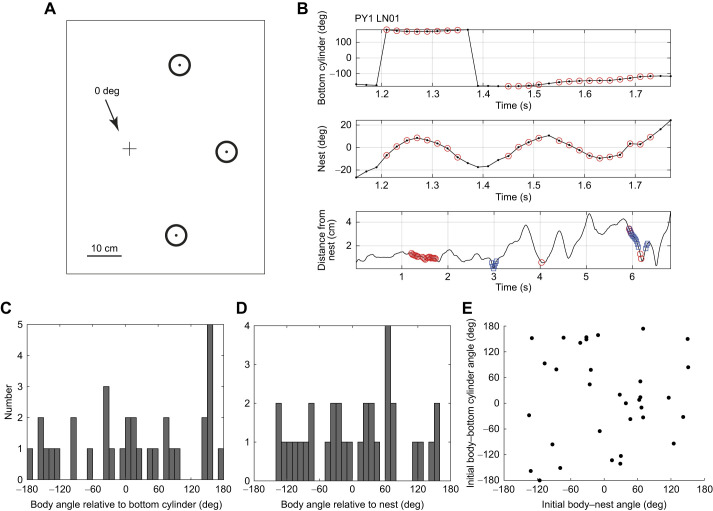
**Initial data.** (A) Layout of cylinders (circles) around the nest (+). The reference direction (0 deg) is shown by the arrow from the nest to the bottom cylinder. The central cylinder is roughly North of the nest. (B) An example of a fixation set within the first 5 cm of a learning flight (bee PY1 LN01). Each graph plots a parameter of a single bee's first learning flight from the nest over a section of the flight and shows how fixations are selected (see Materials and Methods for details). Top: body orientation relative to the bottom cylinder (0 deg). Middle: body orientation relative to the nest. Bottom: whole flight showing the bee's distance from the nest. Blue indicates when the bee faces the bottom cylinder, red indicates when it faces the nest. (C) Bees' body angle relative to the bottom cylinder on the first frame of the videorecording (*n*=33 bees). (D) Bees' body angle relative to the nest on the first frame of the videorecording. (E) Scattergram of bees' body angle relative to the bottom cylinder (as in C) versus body angle relative to the nest (as in D).

The bumblebee, *Bombus terrestri*s, recorded out of doors often faces its nest while pointing in a preferred compass direction – mostly North. Sometimes, the preferred direction is relative to objects in the immediate nest surroundings (Hempel de Ibarra et al., 2009), as in the solitary wasp *Cerceris* sp. (Zeil, 1993a). The tendency to face preferred objects was more marked in the present analysis of learning flights that were recorded in a bare greenhouse with three black cylinders arranged near the nest (Fig. 1A) and where solar cues may be weaker. Bumblebees in this situation tend to face in the rough direction of the bottom cylinder, indicated by the arrow in Fig. 1A (Robert et al., 2018). It cannot be dismissed that this choice of cylinder is influenced by a non-uniform light distribution in the greenhouse. Indeed, Vollbehr (1975) emphasised the importance of the position of the sun in the honeybees' first orientation flight.

In ants, the amount of nest facing depends on the environment in which a species lives. Nest facing lasts longer when the nest is located within vegetation than it does for species with nests in bare surroundings, suggesting that the duration of nest facing may depend on what needs to be learnt about visual features near the nest (Fleischmann et al., 2017).

Bumblebees nest in holes in the ground that are often abandoned by other animals and are quite commonly in undergrowth. Their learning flights have two phases: an initial phase in which the bee flies low to the ground and is close to the nest, and a later phase when it flies higher and further from the nest in a sequence of loops in which it flies towards and away from the nest (Linander et al., 2018; Lobecke et al., 2018; Philippides et al., 2013). Nest facing is prominent throughout the flight (Philippides et al., 2013).

Our present focus is on the start of the initial phase and what happens when bees fixate their nest on their first learning flight. Because colonies of *Bombus terrestris* are available commercially, with new workers emerging from their pupae, we know that the bee's first recorded learning flight is the first time that it sees the visual surroundings of their nest.

How do insects leaving their nest with no knowledge of the landscape around the nest manage to face it? Path integration (Mittelstaedt and Mittelstaedt, 1980; for reviews, see Heinze et al., 2018; Honkanen et al., 2019) contains the required information. It holds a vector of an insect's current direction and distance to the nest throughout a learning walk or flight. It would thus enable an insect to turn and face its nest at will.

Studies by Wehner and colleagues (Wehner et al., 2004; Müller and Wehner, 2010) indicate that the desert ant *Cataglyphis bicolor* uses path integration to face and return to its nest during learning walks. Further evidence has come from applying an artificial magnetic field to ants engaged in learning walks in their natural surroundings. When the direction of the field was shifted, the ants responded by facing towards their nest in the perceived magnetic direction (Fleischmann, et al., 2018). Path integration, rather than visual knowledge of the surroundings, must mediate the ant's ability to face its nest.

The present paper in part echoes earlier work (Collett et al., 2013) that described an interaction between bees' facing the nest and pointing their body in favoured orientations. The work reported now shows that facing the bottom cylinder (Fig. 1A) is most pronounced at the very start of the flight during the bee's first fixation of the nest. We also give an account of the conjunction between fixating the nest and the bottom cylinder in terms of path integration. It was also noted earlier that straight segments of flight occur in preferred compass directions (Collett et al., 2013). A straight segment of controlled flight in a set direction now turns out to be a prelude to the preferred coincidence between nest fixation and body orientation.

First, we examine the precision with which bumblebees face the nest. Second, we detail the relationship between fixating the nest and facing the bottom cylinder. Third, we describe the flight manoeuvre that leads bees to face the nest in a preferred geocentric direction.

## MATERIALS AND METHODS

Experiments were conducted in a greenhouse (8×12 m floor area) at the Streatham campus of the University of Exeter, UK. Bumblebees, *Bombus terrestris audax* (Linnaeus 1758), from commercially reared colonies (Koppert, Haverhill, UK), were marked individually with coloured number tags. The colony was placed under a table, the ‘nest table’, and we recorded the flights of worker bees as we allowed them to leave their nest, one at a time, through a hole in the centre of the table. Three black cylinders (17×5 cm) were placed in a 120 deg arc around the nest hole with their centres 24.5 cm from the hole (Fig. 1A). Another table with a feeder and the same arrangement of cylinders was placed 5 m from the nest table. Both tables were covered with white gravel.

The behaviour of bees leaving the nest and in some cases on their return after feeding was recorded continuously during the experiments at 50 frames s^–1^ with video cameras (Panasonic HC-V720, HD 1080p) that were hung 1.35 m above each table and captured an area of about 70×90 cm in an image of 1920×1080 pixels.

### Experimental procedure

A bee colony, kept indoors overnight, was taken every morning to the greenhouse. The box containing the colony was placed beneath the nest table with the nest-box connected to a hole in the centre of the table via a series of transparent tubes used to control the exits and re-entrances of individual bumblebees from the nest. This arrangement has the side benefit of exposing bees to daylight for a few days before they are first released and can view the nest surroundings. We found that this prior exposure to daylight improves the bees' readiness to leave the nest and fly around the greenhouse.

All the bees contributing to this study were recorded on the first occasion that they left their nest. At the end of their learning flight from the nest, they flew around the greenhouse before being caught and placed on the feeder. Recordings of flights come from Robert et al. (2018) (*N*=18 bees) and from an unpublished study conducted by authors of this paper (*N*=15 bees) that followed almost the same procedure. The difference is that a purple ring surrounded the nest in the first study but not in the second one. The two sets of data were analysed in the same way. Most of the flights were recorded in the afternoon.

### Data analysis and statistics

All videos were examined and clipped with video-editing software (Adobe CS6). We discarded the few flights in which bees landed during the learning flight or flew directly away from the nest. Bees tended to walk a little in an erratic manner when first leaving their nest. We began analysis when the bees started their flight and stopped analysis once the bees had travelled 5 cm from the nest. The positions and body orientations of the bees were extracted from the video recordings using custom-written code in Matlab R2021b. The Matlab script allows corrections by hand, ensuring orientations are accurate to ∼5 deg (details in Hempel de Ibarra et al., 2009). We analysed the bee's body orientation relative to the nest and also its body orientation relative to its surroundings. For the latter, the coordinate system had its origin at the nest with 0 deg the direction of the line from the nest to the bottom cylinder (Fig. 1A).

A single bout of nest facing was mostly quite short, but it could last for almost a second. We use the term ‘fixation’ for a bout of nest facing in which the bee faced the centre of the nest with a precision of ±10 deg for at least 4 frames (i.e. 80 ms). The 80 ms minimum duration of a fixation comes from the duration of plateaus in the staircase of saccades and plateaus seen in bumblebee head movements (Riabinina et al., 2014). If just a single frame within the fixation lay outside the limit of ±10 deg, the frame was included as part of the fixation. To work with separate fixations, we only included those fixations that had an interval of at least 200 ms between the end of one fixation and the start of the next one.

Fig. 1B gives an example of the exclusion of a fixation. The top panel shows the orientation of the bee's body relative to the line from the nest to the bottom cylinder (Fig. 1A). Red circles are frames in which the bee faced the nest (±10 deg). Because the two putative fixations are separated by only 80 ms, one of the two potential fixations was rejected. The second longer 15 frame fixation was the one selected. The middle panel gives the orientation of the bee's body relative to the nest over the same time interval. The single frame outside the fixation at ca. 1.52 s was accepted as part of the fixation. Note its proximity to 10 deg. The longer period of frames outside the fixation at 1.4 s was excluded. The bottom panel is of the whole flight, showing the bee's distance from the nest. Blue indicates when the bee faces the bottom cylinder and red again shows when the bee faces the nest. The bee's first nest fixation does not coincide with the bee facing the bottom cylinder, whereas in the last fixation (around *t*=6 s), nest and cylinder facing overlap.

To determine whether fixations were longer than one might expect by chance, we performed a Monte Carlo analysis with 100,000 repetitions. For each repetition, the frames of all the learning flights over the analysed distance (0–5 cm) were randomised. Fixations were then extracted from all the randomised frames. Statistical tests on the data, including the Mann–Whitney *U*-test, were performed in R version 4.2.1 and in Matlab R2021b using the CircStat tool box (Berens, 2009). The *V*-test with a predicted direction told us whether a distribution was or was not randomly distributed relative to the predicted direction. We also give circular means and the value of the vector **R**.

## RESULTS

On take-off – the first recorded frame of the flight, the bees' body orientation relative to the nest and to the bottom cylinder were very variable (Fig. 1C,D). Initially, there was no obvious relationship between these variables (Fig. 1E). A relationship only emerged later once the bees fixate the nest.

### Nest facing at the start of learning

**Fig. 2.**
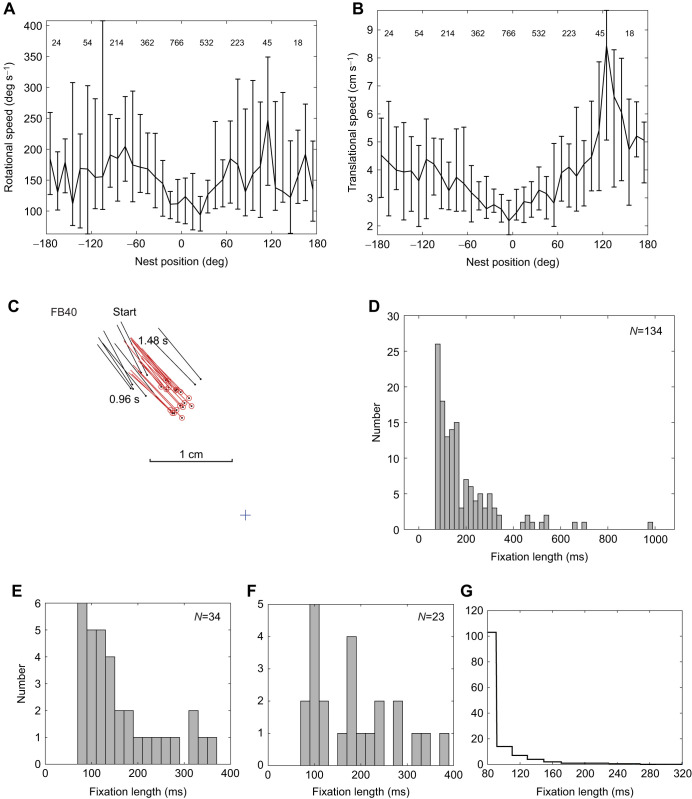
**Nest fixations.** (A,B) Median rotational (A) and translational (B) speed of 33 bees during the first 5 cm of their first learning flights as a function of body orientation relative to the nest. Data are accumulated within 10 deg bins with the speed range indicated by bars from the 25th to 75th percentiles of the data in each bin (i.e. the interquartile range). Numbers above the plot show how many frames the bees spent in that bin and emphasise the preponderance of nest facing. (C) Example of nest fixation. Ball and stick give the position of the head and the orientation of bee FB40 during its fixation of the nest every 20 ms. Red indicates the fixation. To show this bee's precision, the boundary limits of the fixation here are ±5 deg. Points outside this limit are black. The cross (+) is placed here and in other figures at the central point of the nest hole. Times give the start and end of the fixation. (D) The duration of all nest fixations (from −10 to 10 deg) of 32 bees. (E,F) Distribution of fixation durations when bees face towards points that are perpendicular to the nest (E, −80 to −100 deg fixations; F, 80 to 100 deg fixations). (G) Distribution of the relative duration of nest fixations when all the frames in which bees faced the nest are extracted and then randomised 100,000 times.

At the start of the flight, bees translate and rotate more slowly when they face the nest than when they look in other directions (Fig. 2A,B). The number of frames that the bees spent in the associated bins (given at the top of the plots) indicates the predominance of nest facing (Philippides et al., 2013). This behaviour led us to explore nest facing in more detail.

### Fixations

Bees often faced the nest in bouts of consecutive frames that we term fixations (defined in Materials and Methods, ‘Data analysis and statistics’). Fig. 2C shows an example of a fixation illustrating that the bee sometimes remains almost stationary while facing the nest. Fixations occurred in all but one of the examined bees (32 out of 33 bees). The durations of fixations had a broad spread with a maximum close to 1 s (Fig. 2D).

We looked for ways to show that these durations are longer than would be expected by chance, given the general prevalence of nest facing. A first attempt was to examine fixations of points perpendicular to the nest and test whether they were shorter than nest fixations. Although there were fewer of these fixations and none were longer than 20 frames (Fig. 2E,F), a comparison of the distribution of 57 perpendicular fixations with the distribution of nest fixations showed no difference in fixation lengths (Mann–Whitney *U*-test: *z*-score=−0.25, *P*=0.40).

The next step was to examine the length of fixations when the data were randomised in a Monte Carlo analysis with 100,000 repetitions (see Materials and Methods, ‘Data analysis and statistics’). Fixations extracted from all the randomised frames were pooled and a plot of the frequency of their relative lengths (Fig. 2G) shows a sharp drop in the frequency of fixations longer than 5 frames. A comparison (Fig. S1) between the real and randomised distributions of fixation lengths, assuming independence between bins, established that the real fixations are significantly longer (Mann–Whitney *U*-test: *z*-score=−2.10106, *P*=0.01786). The same is true for fixations extracted using the same process that was adopted for the randomised results, but before randomisation (Mann–Whitney *U*-test: *z*-score=−1.91162, *P*=0.02807). We conclude that nest fixations are part of the bee's intended behaviour, justifying our focus on fixations in the following sections.

**Fig. 3.**
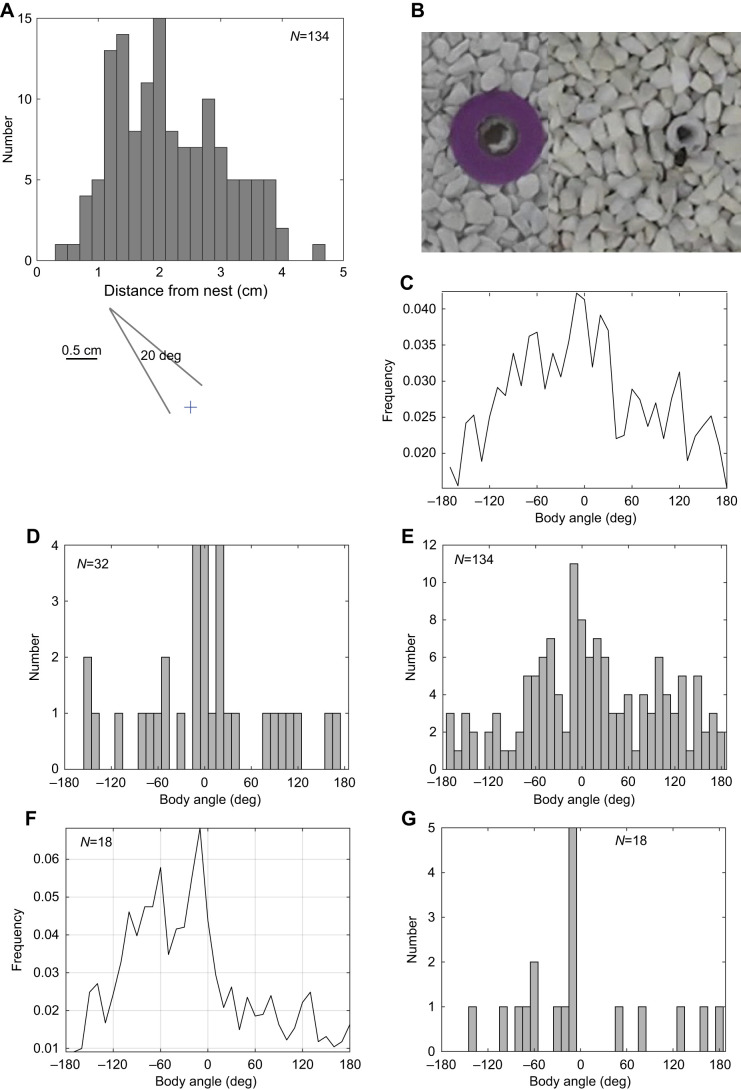
**Body orientation and distance from the nest during nest fixations and body orientation during first return flights.** (A) Horizontal distance of the mid-point of fixations from the nest. The diagram below illustrates the angular span covered by a fixation at 2 cm from the nest. (B) Photo showing nests with and without the purple ring. The diameter of the ring is 5 cm and that of the outer rim of the tube connecting the nest to the outside world is 2.2 cm. (C) Distribution of bees' body orientation relative to the bottom cylinder during all the analysed flights. (D,E) Distribution of body orientation relative to 0 deg at the midpoint of 32 bees' fixations of the nest, for (D) their first fixations and (E) all 134 fixations during the flight. (F,G) Body orientation during first return flights when the bee is within 10 cm of the nest (F, frequency accumulated over all bees; G, peak orientation of each of the 18 bees).

Fixations occurred when bees were close to the nest entrance (median distance=2.1 cm, interquartile range, IQR=1.7 cm; Fig. 3A) so that the immediate nest surroundings (nest exit with and without purple ring; see ‘Experimental procedure’ in Materials and Methods; Fig. 3B) filled much of the bees' ventral visual field. This proximity implies that fixations of the nest centre are precise. A ±10 deg angle of fixation at 2 cm suggests that bees can pinpoint the centre of the nest exit to within ∼0.7 cm (Fig. 3A).

What tells bees that are unacquainted with their surroundings to lower their rotational and translational speeds when facing the nest and so generate a fixation? In the Discussion, we argue why bees are likely to behave like ants (Wehner et al., 2004; Müller and Wehner, 2010; Fleischmann et al., 2018) and rely on path integration to fixate the nest. One benefit of using path integration is that it allows bees to set the orientation of their body when facing the nest.

### Preferred body orientations during nest fixations and on return flights

The bees' overall preferred viewing direction during the flight was towards the bottom cylinder (Fig. 3C). To test whether this preference might be stronger during fixations, we measured the direction of the line from the nest to the bottom cylinder at the midpoint of each fixation. Peak body orientation was 0 deg. The effect was pronounced on the bees' first fixations of the flight, but less so if all 132 fixations of the 32 bees were included (cf. Fig. 3D and E). Circular statistics applied to these distributions gave a circular mean of −3.7 deg, **R**=0.39 for the first fixations and 11.6 deg, **R**=0.25 for all fixations. The *V*-test with a prediction of 0 deg indicated that body orientations are not distributed uniformly around 360 deg (first fixations eval=12.54, *P*<0.001; all fixations eval=24.11, *P*<0.001). We also examined the bee's body orientation on the first occasion that it faced the nest during its flight (Fig. S2). These occasions were separated into those which were at the start of a nest fixation (≥80 ms) and those which were shorter than a fixation. In both cases, the bees' distance from the nest peaked at ca. 1–2 cm from the nest. When the first bout of nest facing was a fixation, bees seemed more likely to face the bottom cylinder than if the nest facing lasted less than 80 ms. Brief incidents of nest facing may just be part of a turn and not implemented through path integration.

We then tested whether learning the view stored when fixating the nest might help guide the bees’ later returns to the nest. For 18 of the bees from Robert et al. (2018), there were matching return flights to the nest after the bees' first visit to a flower. We extracted the bees' body orientations during their first return flight to the nest, starting once they were within 10 cm from the nest and stopping when they reached 1 cm from the nest. Over this last section of the return, the distribution of body angles peaked at −10 deg, just to the left of the bottom cylinder, but with a wide scatter (Fig. 3F).

The peak and the scatter were reflected in the bees' individual performance (Fig. 3G). For each bee, we took the peak value of its body orientation during its return. The *V*-test, performed with a prediction of 0 deg on the distribution of peak values, was significant (eval=5.54, *P*<0.04; **R**=0.38, circular mean=36.74).

### Reaching a coincidence between nest fixation and preferred body orientation

**Fig. 4.**
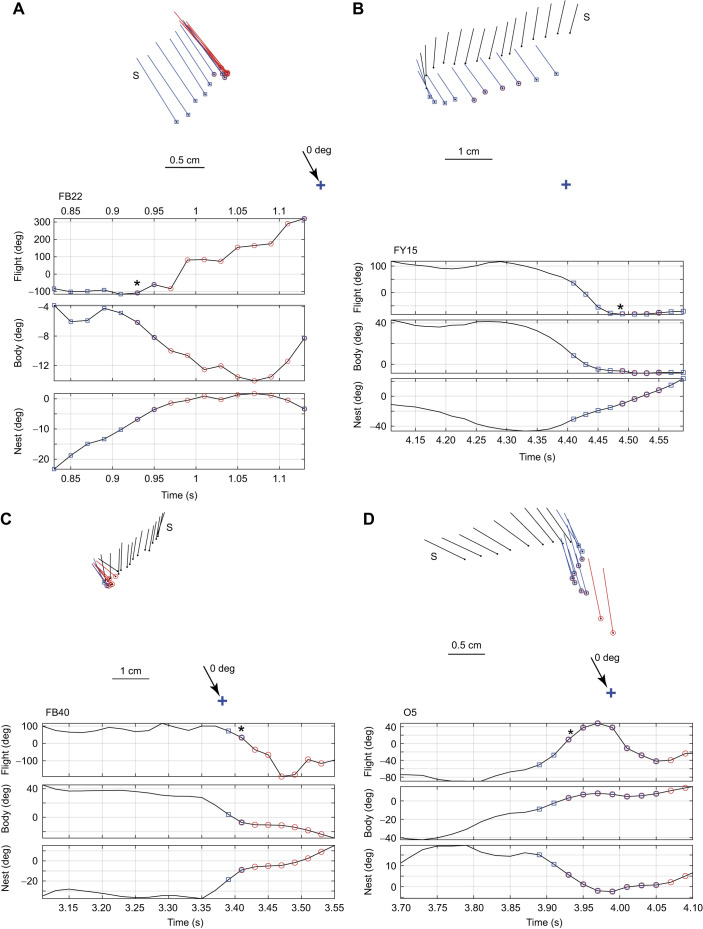
**Translational scans prior to simultaneously facing the nest and the bottom cylinder.** (A–D) Flight paths of the scans and fixations of four bees (FB22, FY15, FB40, O5). The top of each panel shows the bee's flight path. S indicates the start of the scan. The ball and stick representing the bee on each 20 ms frame is red when the bee faces the nest (±10 deg) and blue when it faces the bottom cylinder (±10 deg). Time plots of the flight paths give, from top to bottom: bees' flight direction and body orientation, both relative to the line from the nest to the bottom cylinder; and the body orientation relative to the nest. Asterisks mark the start of coincidence.

**Fig. 5.**
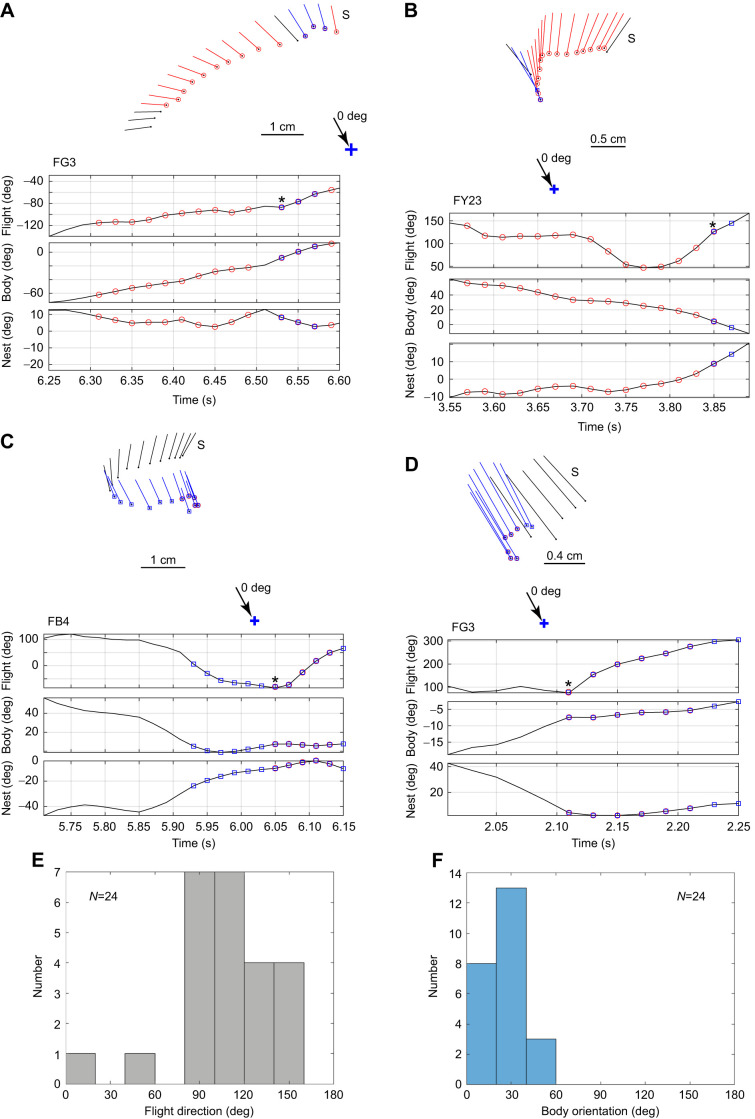
**Translational scans prior to simultaneously facing the nest and the bottom cylinder.** (A–D) Four more examples of translational scans (bees FG3, FY23, FB4). Details as in Fig. 4. (E,F) Histograms of plots of absolute values of flight direction (E) and body orientation (F) relative to the line from the nest to the bottom cylinder. Data come from the scans of 22 bees. Bin width is 20 deg.

Just before bees faced both the nest and the bottom cylinder, they performed a translational scan that was roughly perpendicular to the line between the nest and the bottom cylinder (Figs 4 and 5). This scan led to the conjunction of facing the nest and the bottom cylinder. There were 24 cases of this conjunction. All were preceded by a translational scan. The eight examples in Figs 4 and 5 show some of the variety of flight patterns that occurred. In 15 cases, the body reached the preferred body orientation of pointing at the bottom cylinder (0 deg) before nest facing occurred (e.g. bee FB22, Fig. 4A). In 7 cases, the order was reversed (e.g. FY23, Fig. 5B). In 2 cases, bees faced the nest and the bottom cylinder at the same time (e.g. bee FG3, Fig. 5D).

To obtain an estimate of flight direction and body orientation during 24 scans, the values of the flight parameters were extracted at the midpoint of each scan. With the signs of the angles ignored, the distributions of flight direction and body orientation were almost perpendicular to each other (Fig. 5E,F). With the *V*-test applied to body orientation, a prediction of 0 deg was significant (eval=21.2, *P*<0.0001; **R**=0.98, circular mean=23.39 deg). But with a prediction of 90 deg, the values were at chance level (eval=−0.50, *P*=0.56). For flight direction, the relationships reversed. The *V*-test with a prediction of 90 deg was significant (eval=20.16, *P*<0.0001; **R**=0.85, circular mean=108.01 deg); but with a prediction of 0 deg, the values were not significant (eval=−6.30, *P*=0.97).

## DISCUSSION

The data here add to what we already know about learning flights in *Bombus terrestris* by showing what happens at the start of a bee's first learning flight when it is unfamiliar with the visual surroundings beyond its nest. Despite the bees' ignorance of the outside world, they have a strong propensity to face the bottom cylinder (Fig. 1A) during their first fixation of the nest (Fig. 3D), supporting a functional link between nest fixation and facing that cylinder. Through the rest of the initial phase of the flight, there is also a tendency to point at the bottom cylinder, but it is much weaker (Fig. 3C; Robert et al., 2018).

It is unlikely that bees are facing the sun, as honeybees do when they hover in front of the hive during their first orientation flight (Vollbehr, 1975). The bottom cylinder is roughly 60 deg East of North and almost all the learning flights were recorded in the afternoon. It would nonetheless be worthwhile to examine the bees' behaviour with the array of cylinders rotated by, say, 60 deg.

The bees adopt the same body orientation on their return flights when they are close to the nest. A similarity between learning and return flights in bumblebees is also found in more natural outdoor settings (Hempel de Ibarra et al., 2009; Philippides et al., 2013; Collett et al., 2013) and in other hymenopterans (Zeil, 1993b). A similarity in flight manoeuvres between learning and return flights allows a returning insect to match its stored views to what it currently sees and so approach its nest.

We suggest that the bees' fixation of their nest is mediated through path integration, as it is in ants (Wehner et al., 2004; Müller and Wehner, 2010; Fleischmann et al., 2018). It is difficult to imagine what other mechanism would allow bees to set their body orientation in a favoured direction while fixating the nest. Were bees to store a randomly directed view of the surroundings of the nest at the start of a learning flight, they could fly towards the nest, but would need extra information to set a particular approach direction. Similarly, bees would not be sure of storing a nest-directed view if their only constraint during learning was to face the bottom cylinder.

**Fig. 6.**
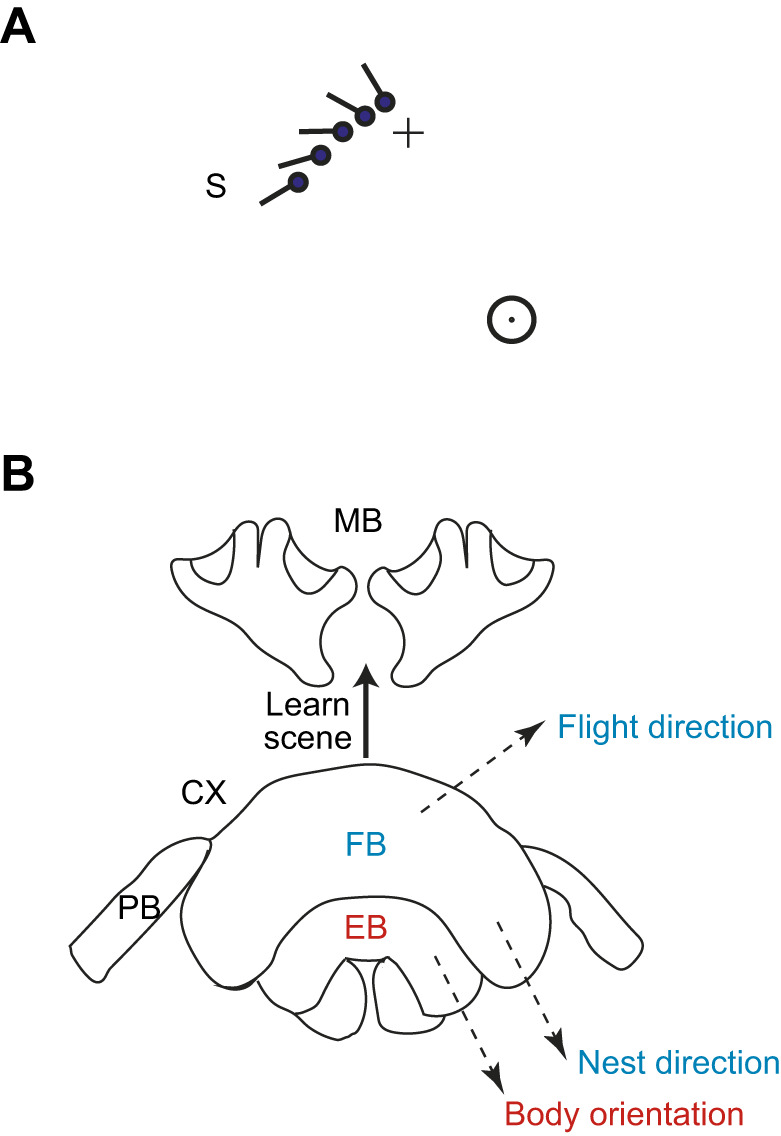
**Translational scan and sketch of the central complex and mushroom body with suggested interactions between the two structures.** (A) Sketch of the way that a translational scan perpendicular to the line between the nest and bottom cylinder can aid the conjunction of nest facing and the bee's preferred facing direction – a time when bees may memorise the scene around the nest. Stick and ball signify the orientation and head of the bee. S indicates the start of the scan. The figure shows a scan that might occur if the nest is fixated before the conjunction. Rotation would be slower for scans in which the bottom cylinder is fixated first. (B) Diagram of the central complex (CX) and mushroom body (MB) (EB, ellipsoid body; PB, protocerebral bridge; FB, fan-shaped body). Learning is likely to occur in the MB when body orientation is 0 deg as encoded in the EB, and the bee at the same time faces the nest as computed through path integration in the FB. Flight direction is also elaborated in the FB.

Lastly, we have learnt that prior to the conjunction of nest fixation and facing the bottom cylinder at 0 deg, the bee controls its flight direction. It moves in a direction that is approximately 90 deg from zero. This translational scan (Figs 4 and 5) helps the bee reach the conjunction between nest fixation and its preferred viewing point in the nest surroundings. Sometimes, body orientation is close to zero during the scan. In this case, the conjunction will be reached when path integration tells the bee that it is facing the nest. Sometimes, bees face the nest while scanning and can stop scanning when they reach their preferred body orientation (shown schematically in Fig. 6A).

### Flight parameters and the central complex in the insect brain

A bumblebee's body orientation within its surroundings, its fixation of its nest, and its flight direction contribute in an organised way to its ability to memorise views during learning flights. These three parameters of its flight are most likely controlled by the central complex (Fig. 6B). In *Drosophila*, the direction in which a fly faces is encoded spatially within the ring-like ellipsoid body (Seelig and Jayaraman, 2015). The ring consists of 8 tiles with each sensitive to a particular heading direction. Only a single tile is active at any time and the fly points in the direction encoded by the active tile. Visual input to the ring is carried by a population of ring neurons with inhibitory processes that connect all the other tiles. In consequence, the active ring cell determines which point in the scene attracts the fly's attention (Fisher et al., 2019). This mechanism is well suited to enable a bee to face the bottom cylinder.

Nest fixations most likely involve path integration and much of the circuitry supporting path integration resides within the fan-shaped body of the central complex (Stone et al., 2017). A further actor, flight direction, is needed to generate the bee's scan. In *Drosophila*, flight direction, independently of body direction, is also computed within the fan-shaped body (Lu et al., 2022; Lyu et al., 2022). Our data suggest that the three flight parameters are closely coordinated in generating the scan that precedes a nest fixation (Figs 4, 5A,B). We have sketched this outline of events in the central complex to emphasise that it would be interesting to know how the central complex might implement the coordination of the three flight parameters.

While the central complex controls the bee's movements, the views of the nest that bees acquire are most likely stored within a structure known as the mushroom body (Fig. 6B). Lesions to the mushroom body disrupt visual navigation in ants (Buehlmann et al., 2020; Kamhi et al., 2020). Furthermore, lesions to the mushroom body of cockroaches disrupt the insect's ability to locate a familiar place (Mizunami et al., 1998; Strausfeld, 2012). There may be an apposite connection in *Drosophila* between the central complex, which organises the direction in which the insect looks, positioning it to obtain a suitable view, and the mushroom body, which stores that view. Zolin et al. (2021) report a relationship between walking direction and dopamine activity within the mushroom body. Such a partition of tasks between the central complex and the mushroom body (Fig. 6B) suggests that if the input from the mushroom body to the central complex were to be experimentally interrupted, a bee may be able to generate the motor programme of a normal learning flight, but without learning the scene in a useful way.

## Supplementary Material

10.1242/jexbio.245271_sup1 Supplementary information
