# Supplementary material for: How bumblebees coordinate path integration and body orientation at the start of their first learning flight
Source: J Exp Biol. 2023 Apr 21;226(8):jeb245271. doi: 10.1242/jeb.245271 (PMC10263152; doi:10.1242/jeb.245271)
Supplement: Supplementary information [file jexbio-226-245271-s1.pdf]

A

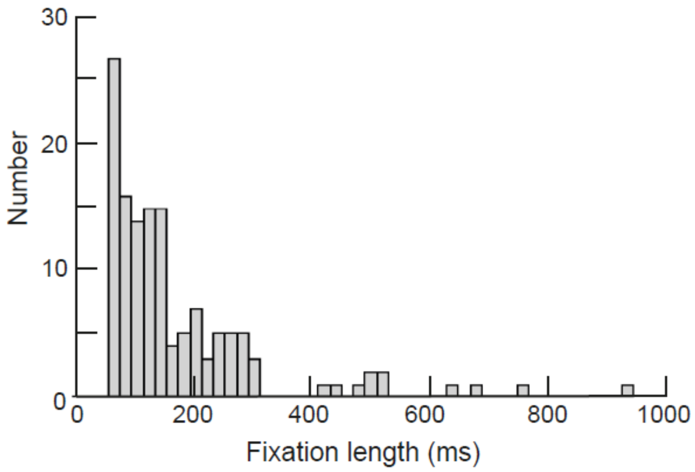

B

Fixation length (ms)	80	100	120	140	160	180	200	220	240	260	280	300	320	340	360	380	400
No: fixes: Real_a	26	18	13	14	15	3	7	6	4	5	3	5	3	2	0	0	0
Real_b	43	14	15	15	4	5	7	3	5	5	5	3	0	0	0	1	1
Random	103	14	7	4	2	1	1	1	0.4	0.2	0.2	0.1	0.1	0	0	0	0

Fig. S1. Testing whether randomisation of frames across all flights leads to fewer long nest fixations than is observed in the data from real bees

A. Length of fixations extracted automatically from 32 learning flights. Plot is very similar to the corresponding plot extracted manually (Fig. 2D), the major difference being that criteria of 200ms separation was not applied .

B. Testing whether real fixations are longer than those occurring if all the frames from all the analysed nest-learning flights are randomised 100,000 times. All rows are normalised to a total of 134 fixations. The analysis stops when fixation duration reaches 400ms and the frequency of further random fixations is well below 1.

Journal of Experimental Biology • Supplementary information

The diagram has four rows. The top row shows the length of the fixations from 80 to 400ms. The next three rows give the number of occurrences of each fixation length. First row: fixation lengths as shown in Fig 2D; second row: fixation lengths as shown in in Fig. S1A; third row: fixation lengths after randomisation A comparison between the real and randomised distributions of fixation lengths, assuming independence between bins, establishes that the real fixations are significantly longer (Mann-Whitney U test: z-score = -2.10106, p= 0.01786). The same is true for fixations extracted using the same process that was adopted for the randomised results, but before randomisation (Mann-Whitney U test: z-score = -1.91162, p=0.02807). Randomisation thus leads to more short fixations and fewer long fixations.

Journal of Experimental Biology • Supplementary information

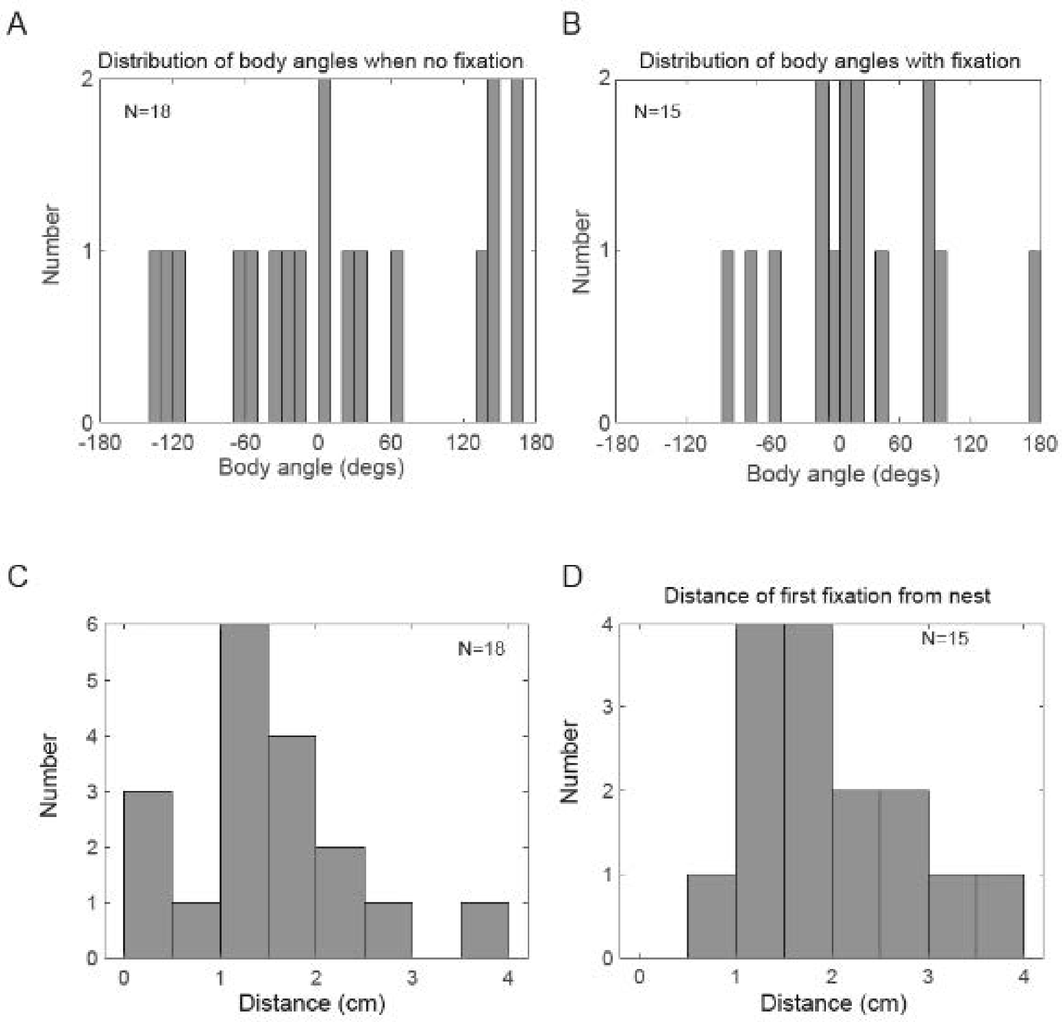

Fig. S2. First frame of learning flights in which bees face the nest.

A. Distribution of body angles during first nest facing frame when that frame is not part of a fixation.

B. Distribution of body angles during first nest facing frame when that frame is the start of a fixation.

C, D. Distribution of distances from the nest of the first nest facing frame. C, frame is not part of a fixation. D, is part of a fixation.

Note: Distribution in B is more focussed on bottom cylinder than is distribution in A.
